# Experiences and Perceptions Within a Co-Created Drone Transport Initiative With Rural First Nation and Non–First Nation Communities: Semistructured Interview Study

**DOI:** 10.2196/82720

**Published:** 2026-05-29

**Authors:** Audrey Warner, Sandy Lee, John Pawlovich, Terri-Leigh Aldred, Michael Allard, Dave Christie, Alison James, Dave Kang, Kimia Nouhi, Larry D Lynd, Robert Michell, Adam Patrick, Edward Ratnarajah, Anurag Singh, Sarrah Storey, Femke Hoekstra

**Affiliations:** 1 Department of Family Practice, Faculty of Medicine, University of British Columbia, Vancouver, British Columbia, Canada; 2 Rural Coordination Centre of BC, Vancouver, British Columbia, Canada; 3 Carrier Sekani Family Services, Prince George, British Columbia, Canada; 4 First Nations Health Authority, West Vancouver, British Columbia, Canada; 5 National Collaboration Center of Indigenous Health, Prince George, British Columbia, Canada; 6 Department of Pathology and Laboratory Medicine, Faculty of Medicine, University of British Columbia, Vancouver, British Columbia, Canada; 7 Village of Fraser Lake, Fraser Lake, British Columbia, Canada; 8 School District 91 Nechako Lakes, Vanderhoof, British Columbia, Canada; 9 Sobeys, Vancouver, British Columbia, Canada; 10 Faculty of Pharmaceutical Sciences, University of British Columbia, Vancouver, British Columbia, Canada; 11 Centre for Health Advancing Health Outcomes, Vancouver, British Columbia, Canada; 12 Providence Health Care Research Institute, Vancouver, British Columbia, Canada; 13 Stellat'en First Nation, Stellako, British Columbia, Canada; 14 Thrive Health, Vancouver, British Columbia, Canada; 15 Division of Nephrology, Faculty of Medicine, University of British Columbia, Vancouver, British Columbia, Canada; 16 Division of Medical Sciences, University of Northern British Columbia, Prince George, British Columbia, Canada; 17 Centre for Chronic Disease Prevention and Management, Faculty of Medicine, University of British Columbia, 1088 Discovery Avenue, Kelowna, British Columbia, V1V 1V7, Canada, 1 250 807 8876; 18 Division of Social Medicine, Department of Medicine, University of British Columbia, Vancouver, British Columbia, Canada

**Keywords:** rural, technology, health care innovation, community engagement, health care access, First Nations, Indigenous

## Abstract

**Background:**

People living in rural areas of British Columbia experience inequities in access to health care that impact health and well-being. Since time immemorial, Indigenous peoples have had a holistic understanding of health and wellness, and knowledge of healthful ways of living. However, in a rural context, Indigenous peoples contend not only with inequitable access to health care, but also with the historical and ongoing impacts of colonization. Virtual health innovations enable access to care closer to home, yet the need for diagnostic tests and essential medicines remains limited by supply chains. In this context, transport of medical supplies by drone offers a promising solution and has the potential to improve access to health care in rural and First Nations communities in British Columbia.

**Objective:**

The Drone Transport Initiative is a co-created health care innovation project between the Stellat’en First Nation, the Village of Fraser Lake, the University of British Columbia, and other health system partners that investigated the feasibility of drones to transport medical supplies in a rural British Columbian context. This analysis aimed to understand the experiences and perceptions of the Project Team, people directly involved in the Initiative.

**Methods:**

Twenty members of the Project Team, including representatives from the Steering Committee and Operational Team having diverse roles within the project, participated in semistructured interviews. Eleven interviews were conducted either individually (n=5), in pairs (n=5), or in a group of 5 (n=1). Interviews were audio-recorded, transcribed verbatim, and anonymized. A reflexive thematic analysis was conducted to understand the Project Team’s experiences and perceptions of the project. Initial results were shared with coauthors, including project cosponsors and community members, to interpret the findings and formulate discussion topics.

**Results:**

Participants generally expressed positive experiences from being part of the Drone Transport Initiative, despite remarking on various challenges. Major themes derived from the analysis centered upon (1) building respectful and trusting relationships, (2) mutual benefits that enabled effective and engaged partnerships, (3) meaningful community engagement that facilitated community acceptance and ownership of the project, and (4) this project is “the first step of something big.” Themes were further divided into subthemes characterized as processes or outcomes.

**Conclusions:**

In this work, we highlight a health innovation project grounded in relational approaches and partnerships, where rural and First Nations communities are champions of advancing their development goals and co-creating solutions that address key social determinants of health. We contribute to the literature by emphasizing the relational foundation that is necessary at the cutting edge of innovation to co-create, implement, and sustain drone projects with rural and First Nations communities.

## Introduction

Rural areas in Canada comprise 98% of the total land mass and are home to 20% of the population [1]. While definitions of rurality differ, in British Columbia’s health care context, rural areas are classified based on proximity to larger health centers and the number of practicing physicians in the area [2]. It is well known that rural populations face inequities in health care: a result of fewer providers, less health infrastructure, and decreased access to specialized services [3]. Rural residents must often travel vast distances to access care, resulting in additional financial costs termed the “rural tax” as well as concomitant social and emotional burdens [4]. These factors highlight geographical location as a key social determinant of health and well-being in a Canadian context.

Indigenous peoples, including First Nations, Inuit, and Métis peoples, across Turtle Island—colonially known as North America—have practiced healthy lifestyles for generations and maintain a holistic understanding of health and wellness [5]. However, historical and persistent colonial forces have intentionally dispossessed and displaced Indigenous peoples from their lands and cultures, and undermined Indigenous worldviews, ways of knowing, and right to self-determination [6]. This historical and ongoing harm has created significant consequences for the health and well-being of Indigenous peoples today. Moreover, Indigenous peoples in rural and remote communities contend with barriers to health at the intersection of colonialism, geographical inequities, and other inequities [5,6]. The need for systemic change is increasingly being recognized within government and nongovernment institutions, academia, and in society at large [7-9]. However, addressing the systemic and structural roots of health inequities is an ongoing task.

Virtual health innovations can supplement in-person health care and address access gaps, especially in rural and remote communities in British Columbia and enable First Nations to access care closer to home [10]. Telehealth services improve access to providers, yet geographical location still impacts access to diagnostic tests and essential medicines. A solution is desperately needed. Drones have been used to deliver health care supplies in multiple contexts globally, to augment the existing supply chain system and enable more timely care [11]. In Canada, drones may complement virtual health services and enable people living in rural areas to access care closer to home. This has the potential to improve overall health and well-being. However, there is currently a lack of research within a North American context focusing on real-world implementation projects that use drones in rural and Indigenous communities, and particularly, the corresponding relational considerations [12].

The Drone Transport Initiative (DTI) is a health care innovation project co-created by the Stellat’en First Nation, the Village of Fraser Lake, the University of British Columbia (UBC), and other health system partners. Phase I of the DTI began during the COVID-19 pandemic when a province-wide state of emergency was declared and transportation was restricted. Many First Nations in British Columbia were locked down, which exacerbated existing inequities in access to health care. At this time, the confluence of political will, regulatory agility, community motivation, and funding opportunities during the COVID-19 pandemic created conditions for rapid innovation. Conceived with the intention to showcase health care applications of drones in resource-constrained settings in British Columbia, the DTI received funding for a 1-year demonstration project. While federal regulations at the time did not permit the transport of patient medications, the project aimed to examine the partnerships necessary to integrate drone technology into communities through the transport of mock medical supplies. In doing so, the project had the potential to provide evidence for regulatory changes, inspire government support, and enable ongoing co-creation with communities. Our team analyzed interview data from people involved in the DTI to identify key facilitators and inhibitors to implementation (K Nouhi, unpublished data). However, analysis of facilitators and inhibitors alone provides only a partial perspective. Gaining a deeper understanding of the broader experiences and perceptions of those involved is also critical for supporting the DTI’s sustainability and is the focus of the analysis herein. This particular line of inquiry lent itself to a reflexive thematic analysis of interviews to elicit the unique perspectives of the DTI Project Team.

## Methods

### Project Team Structure

The DTI Project Team comprised the Steering Committee (SC) and Operational Team (OT). The SC advised on project direction and included sponsors, the project manager, and representatives from partner organizations with leadership roles. The OT included those who were directly involved in day-to-day operations and oversight. Some were hired by UBC and others were hired by the drone technology provider.

### Description of Host Communities

Since time immemorial, Stellat’en (the people of Stella) have lived where the Endako and Stella Koh rivers meet, and the Stellat’en First Nation has a population of about 250 people living on the Reserve [13]. The Village of Fraser Lake, approximately 7 km to the east, has a population of about 1000 people and is located on the traditional, ancestral, and unceded territories of the Stellat’en and the Nadleh Whut’en First Nations. The two communities are approximately 160 km west of Prince George, the largest city in Northern British Columbia.

### Sampling and Data Collection

**Figure 1.**
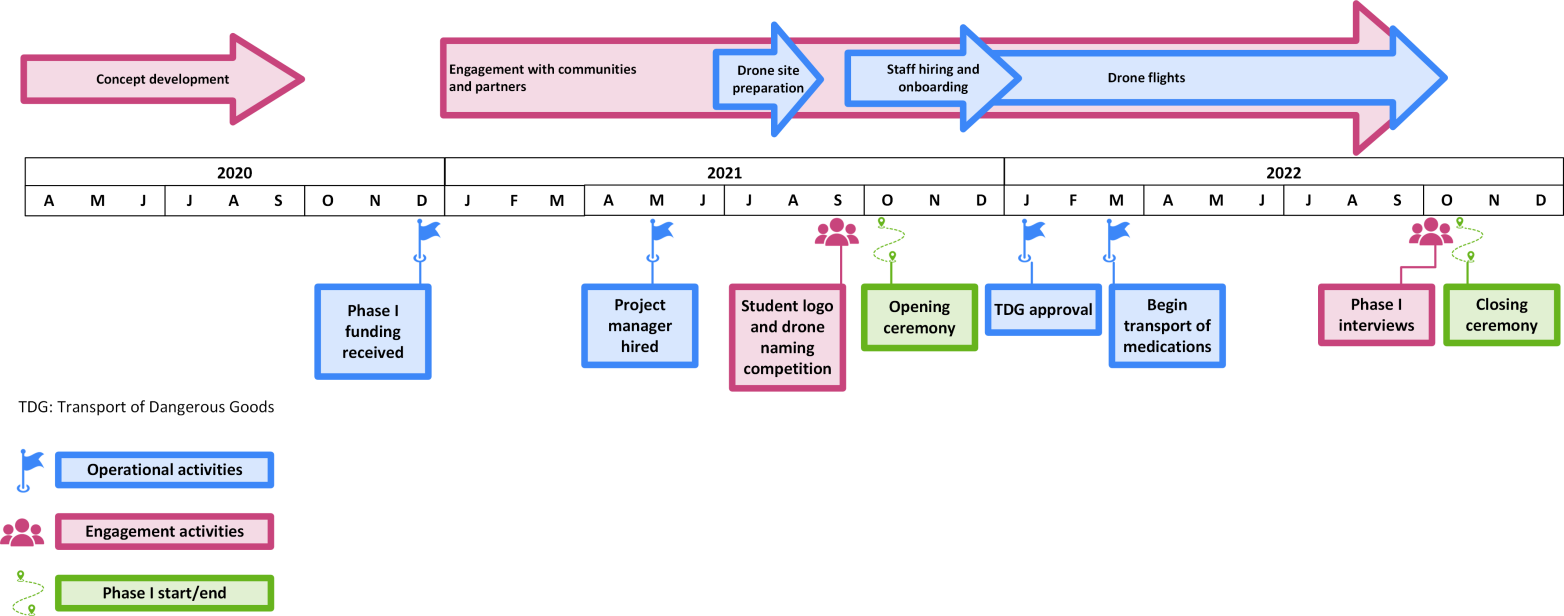
Timeline of key events and activities in Phase I of the Drone Transport Initiative.

All members of the DTI Project Team were invited via email by SL to participate in semistructured interviews. Of the 22 eligible participants, 20 agreed to be interviewed and 2 declined. Semistructured interviews were conducted in-person or over Zoom (Zoom Communications, Inc) by SL in October 2022 (Figure 1). Eleven total interviews were conducted, individually (n=5), in pairs (n=5), or in a small group of 5 (n=1), and lasted between 20 and 60 minutes. All interviews were audio-recorded, transcribed verbatim, and anonymized with numerical IDs. The anonymized transcripts are securely stored on UBC’s OneDrive, and only the lead authors (AW, SL, and FH) have access to the data. All lead authors who interacted with the data have completed training in the Tri-Council Policy Statement: Ethical Conduct for Research Involving Humans (TCPS 2) and are trained in Ownership, Control, Access, and Possession principles. These data have not been returned to communities; however, trusting partnerships between communities and UBC Project Team members render UBC’s OneDrive the preferred location for secure data storage. Data remain accessible to communities should they require access in the future. To enhance the readability of results, AW assigned pseudonyms to participants at random and shortened or revised quotes as necessary. Participants whose quotes are included in the results were contacted for approval of their pseudonyms and for the use of their quotes in the final paper. No participants disagreed.

SL had accumulated several years of experience working in public policy for the First Nations Health Authority in British Columbia. Prior to interviews, most participants had completed a short survey based on the Awareness, Desire, Knowledge, Ability, and Reinforcement model of behavior change [14]. The survey context was specific to using drones to transport medical supplies and included questions about partnership development within the project. SL created an interview guide based on survey questions, which was reviewed with community liaisons, project sponsors, the SC, and the receptionist at the Stellat’en First Nation Wellness Center prior to use (Multimedia Appendix 1). The specific survey findings, including group-level responses, are available in Multimedia Appendix 2. We acknowledge that these tools are rooted in Western, Eurocentric approaches to inquiry, yet have aimed to incorporate Indigenous perspectives and approaches to inquiry in this work by seeking input from Stellat’en First Nation members and representatives from First Nations Health Authority (Multimedia Appendix 2).

The DTI took a strength-based approach to the development of interview questions. The interviews aimed to find out from the DTI Project Team “what went well” and “what could have gone better” during Phase I of the project. Members of the Stellat’en First Nation who participated in surveys or interviews were provided with a US $25 gift card to acknowledge their contributions. A temporary, part-time position with opportunities for further training in project evaluation was advertised through the Stellat’en First Nation’s administrative office with the objective of hiring an Indigenous evaluator to conduct interviews. However, the team was unable to fill the position, so interviews were conducted by SL, who knew all participants, having been the DTI project manager since May 2021 (Figure 1). A more fulsome description of the project can be found in Multimedia Appendix 3.

### Data Analysis

This analysis is 1 of 2 that will inform future phases of the DTI (K Nouhi, unpublished data). Using a pragmatic perspective, this study considers the truth as relative to individual experiences, and practical outcomes are considered knowledge [15]. A reflexive thematic analysis was conducted with interview data to understand the perceptions and experiences of those involved in Phase I of the DTI. AW managed and coded data using NVivo 14 (Lumivero). An inductive approach following Braun and Clarke’s 6-step process facilitated the organic development of a narrative [16]. This involved reading transcripts closely to get familiarized with the data and generate codes while staying as close as possible to the data (ie, avoiding interpretations during the early stages of analysis). Codes were grouped into categories that reflected specific topic areas, such as communication and partner engagement. Themes were generated to encapsulate related categories and summarize an overall message.

Throughout the iterative analysis, SL and FH helped clarify ideas, refine, generate, and review the themes. SL and FH also helped AW create subthemes, which were categorized as either processes or outcomes to produce a preliminary narrative. AW, SL, and FH sought feedback from AJ, JP, and MA, whose feedback further guided the analysis. Data were presented to community coauthors AP, DC, SS, and RM to seek essential feedback and ensure the narrative resonated with communities’ experiences. All coauthors were considered “critical friends” and provided essential context and interpretation to preliminary findings based on their direct involvement with the project. The first author (AW) has been transparent in how her positionality shapes the results of this qualitative study (Multimedia Appendix 4). Transparency in reporting was supported by completion of the COREQ (Consolidated Criteria for Reporting Qualitative Research) checklist (Checklist 1).

### Ethical Considerations

In accordance with the regulations of the Tri-Council Policy Statement (TCPS 2) governing research ethics in Canada, this study did not undergo a full ethical review as it was not classified as research [17]. In Canada, program evaluation and quality improvement studies do not fall under the auspices of the TCPS or institutional Research Ethics Boards. Participants provided informed consent for the interview and audio recording. Participation in interviews was voluntary, and everyone received a CAD $25 (~US $18) gift card to acknowledge their contributions.

This paper represents entirely original work. Where work and/or words of others have been used, this has been appropriately cited or quoted, and permission has been obtained where necessary.

## Results

### Overview

We identified 4 themes and 11 subthemes categorized as processes or outcomes (Textbox 1). Processes are steps or activities to achieve an outcome, and outcomes are the results of these processes.

Textbox 1. **Themes derived from the thematic analysis.**

- Respectful and trusting relationships are key to designing and implementing a community drone project
- Opportunities for mutual benefits lead to effective and engaged partnerships
- Meaningful community engagement helps the drone project become embedded into communities
- The drone represents opportunities and potential for rural and First Nation communities in health care and beyond

### Theme 1: Respectful and Trusting Relationships Are Key in Designing and Implementing a Successful Drone Innovation Project

#### Overview

Natalie (SC) shared, “...the human relationships on the ground were really, really, really valuable.” The first theme focuses on the importance of building and strengthening respectful and trusting relationships between project partners. Processes included cultivation of respectful relationships by project champions and flexible and meaningful engagement of involved partners. A perceived outcome was mutual understanding between the involved partners.

#### Process: Cultivation of Respectful Relationships by Project Champions

Participants recognized that building and strengthening relationships with communities and partner organizations was foundational for progress. Natalie (SC) shared, “how important it is to build those relationships because once you build them, you can just keep moving on.”

Participants identified the instrumental role of project champions in advocating for communities and communicating the project’s vision, opportunities, and potential. Champions contributed to interest and confidence in the project from communities and partners alike. They also played a crucial role in promoting a culturally safe environment through training that supported effective collaboration. These actions were essential in cultivating relationships between diverse partners and communities.

Sydney (SC) mentioned that in addition to having prior experience working with First Nations on community projects, “support also from UBC to guide us on the right path” was invaluable, and felt that “it was a great joint effort ... [that] made it easy at the end.”

One participant highlighted Dr John’s role,

*I have to give him a lot of credit because he does fight for our communities, and I believe that if we didn’t have him to speak for us and say how [the DTI project is] necessary, and how it’s needed, it wouldn’t have happened.*

[Spencer, SC]

Likewise, Chief Robert’s invaluable leadership and support of the project were highlighted,

*I was really impressed with Chief Robert and his commitment and dedication to the project. He just really looked committed to it. He seemed to be communicating with his membership, and he just seemed really engaged which is really great to see.*

[Denise, SC]

Denise (SC) also noted the project manager’s contributions, “I think the project ran superbly, thanks to you. You’re exceptional as to how you managed every detail.”

Being endorsed by project champions facilitated the project manager’s ability to build trust with communities, effectively delegate resources at an operational level, and act as a conduit between champions and the OT.

#### Process: Flexible and Meaningful Engagement of Involved Partners

Participants perceived collaboration, commitment, and interdependence of project partners as an important strength of the DTI. There was also a sense of pride in partners and communities coming together to co-create an innovative technological solution to advance the delivery of health services in rural and First Nations communities. Justine (SC) noted, “It was a diverse pocket of people that made this thing work, because it’s so novel, right?” One participant shared a similar view,

All the different levels, all the different types of personalities, I guess, agencies that are involved to make it come together. I thought that was awesome to have all of that.

[Elise, SC]

SC members were engaged at precommittee meetings, which built an understanding of the different partner roles and the project’s vision and goals. Participants indicated that respectful partner engagement is done according to community protocols and requires an understanding of structures and relationship dynamics within organizations and communities.

Initially, meaningful engagement was deduced during analysis from factors that participants highlighted: collaboration, commitment to one another, and respect for individual, cultural, and geographical context. This aligns with definitions of meaningful engagement from other fields, such as spinal cord injury research, where meaningful engagement comprises personal and socially meaningful research, dissemination and/or implementation, and feeling a sense of responsibility to others [18]. Importantly, community coauthors were consulted to develop and approve the following definition of meaningful engagement. In the context of the DTI, meaningful engagement is an approach to planning, implementing, and sharing the project with community members that prioritizes respectful and trusting relationships and centers community values and perspectives.

Some participants experienced personal circumstances, work obligations, or pandemic-related losses that intermittently limited their capacity to engage in the project. Natalie (SC) identified flexible engagement as a way of accommodating partners’ capacities. She noted,

*I appreciated that we could tap out for those reasons. I felt that we were brought in at the beginning and then [the Project Manager] just connected with us when you needed to, when things were moving ahead. I appreciated not being brought along for every stage.*

[Natalie, SC]

Within the project, participants saw value in balancing the achievement of project objectives with respect and empathy for the humans who are involved in the work.

#### Formation of Diverse Relationships Enabled Mutual Understanding

Participants viewed the formation of relationships between diverse partners as a key positive outcome of the project. For example, Denise (SC) shared,

The best thing about the project was the relationships that were formed between [the Village of] Fraser Lake, the Stellat’en First Nation, and our project group.

[Denise, SC]

These relationships supported effective partnerships across different sectors, areas of expertise, and lived experiences by enabling partners to better understand each other and “appreciate the challenges that rural and remote communities have to deal with.*”* Understanding that these challenges stem from geographical location, as well as the historical and ongoing impacts of colonization for First Nations peoples, helped to contextualize the critical importance of projects like the DTI.

### Theme 2: Opportunities for Mutual Benefits Lead to Effective and Engaged Partnerships

#### Overview

The second theme explores the mutual benefits that motivated the continued participation of project partners. Contributing processes included (1) creating opportunities for community development and (2) facilitating career development and benefiting partner organizations. Perceived outcomes were strengthened community capacity and the realization of organizational benefits.

#### Process: Creating Opportunities for Community Development by Hiring Community Staff

Participants identified Dr John’s role in conveying the project’s opportunities for community development and well-being, both in the Stellat’en First Nation and the Village of Fraser Lake. For example, one participant shared,

*Dr. John started articulating what it was going to look like, who the players are at the table, what the vision is, what the economic opportunities are going to be, what the technology is going to look like.*

[Louise, SC]

This helped to affirm the project’s potential. Donna (OT) also shared her positive perceptions of local employment opportunities and noted, “I like that you guys hired out of the First Nations community here, that’s pretty sweet.”

#### Process: Facilitating Career Development and Benefiting Partner Organizations

Participants mentioned the importance of feeling useful within the project. Some OT members felt that using their existing skillset or developing new skills would allow them to make meaningful contributions and feel more engaged in the DTI project. However, some participants did not feel supported by their organization to get more training. Donna (OT) shared that she wanted to “get more training and be a little more useful” but expressed that her organization did not take her seriously or fully empower her to further develop her skillset.

Kate (OT) suggested that improved communication would have helped her to better understand the project’s objectives, feel more engaged, and think innovatively. When referring to the level of engagement she experienced within the project, she shared,

*It wasn’t that bad, because if it was a really big problem and I felt I was completely in the dark, then I would have been like, ‘Hey what’s the purpose here?’ So, it was clear enough that I didn’t have to seek out understanding, but it wasn’t so clear that I could start doing out of the box thinking in my day-to-day.*

[Kate, OT]

For Kate, a directive approach by her organization did not foster engagement or motivation.

While SC members generally perceived that they were engaged appropriately, experiences from OT members were mixed. For OT members, whose roles within the project were not always stimulating, individual-level benefits, such as the opportunity to upskill or grow professionally, may have been leveraged as an additional source of motivation.

#### Outcome: Strengthened Community Capacity and Achievement of Organizational Benefits

Participants perceived strengthened individual and community capacity as a positive outcome of the project. In addition to tangible outcomes such as deliveries of patient medications, employment opportunities, and exposure to technology, SC members were particularly motivated by the opportunity to support their communities in stimulating economic activity. For example, one participant shared that

one of the big priorities was the opportunity to expose some members to new technology and to the employment opportunities. That was real [sic] important.

[Philip, SC]

Louise (SC) also highlighted the positive impact of technology in a rural context, “...to be able to bring this type of technology to a rural area is so beneficial to everybody.”

Both SC and OT members recognized the benefits of this project to organizations of which they were a part. For example, one participant shared that the project allowed her organization

to take a closer look at drone technology and have a lot of learning. It was good for [our organization] to be a leader in the space, looking to innovate on behalf of the patients of Canada.

[Denise, SC]

Likewise, one participant noted that this project had

been successful because the goal was to evaluate if drone transportation was feasible, and to gather metrics to help us build the industry and see how we can scale this going forward.

[Daryl, SC]

### Theme 3: Meaningful Community Engagement Helps the Drone Innovation Project Become Embedded Into the Community

#### Overview

The third theme focuses on the role of meaningful community engagement in facilitating health care innovation projects. A process in this theme was continuous engagement of community members guided by community representatives, and a perceived outcome was community acceptance and ownership of the project.

#### Process: Continuous Engagement of Community Members Guided by Community Representatives

Participants recognized the importance of continuous community engagement throughout the project. Informal conversations and events, including open houses and visits to the drone site by youth, helped to share the project’s aims with community members. Community liaisons provided advice on how to best engage Stellat’en First Nation and Village of Fraser Lake community members and encouraged a focus on youth engagement.

One participant noted that

having the schools do site visits helped create more buzz around what we were doing. And it had some kitchen table talk when the kids went home at night which helped promote the project as well.

[Christopher, SC]

The drone’s flight path was highly visible to communities, making public acceptance a priority. Louise (SC) noted how communities were informed about the project using posters, Facebook (Meta Platforms, Inc), the community newsletter, and an open house. She noted that the drone would affect community members,

…not only Mrs. Smith that lives close is going to be impacted, but Mr. Brown that lives on the other side of town might come down to White Swan Park and walk the dog. And then there’s a drone [and he] wants to know what’s going on.

[Louise, SC]

However, she remarked,

I had zero complaints, zero comments, zero anything on the first day of the drone flying. I was expecting the phone to go off the hook, and nothing. So, I think we did a good job of explaining.

[Louise, SC]

Indeed, maintaining positive public perceptions was important. Philip (SC) joked, “so far nobody is taking a shotgun to the drone.”

#### Outcome: Community Acceptance and Ownership of Co-created Health Care Innovation Project

Participants highlighted the role of the community in determining their own priorities and interests. One shared that

acceptance by the community itself to allow [the project] to go ahead and not to have too [many] issues with it, that was a good thing.

[Elise, SC]

Louise (SC) mentioned that some community members went to watch the drone fly and perceived this level of interest to be evidence that community engagement was done well.

I think we did have a pretty good community engagement because in the first two weeks of operation it was like a drive-in theater down there. There were vehicles parking just to watch this thing go, alright? Which was pretty neat to see.

[Louise, SC]

### Theme 4: The Drone Represents Opportunities and Potential for Rural and First Nation Communities in Health Care and Beyond

#### Overview

The final theme explores participants’ visions of what the future of drones might look like. A process involved maintaining the vision during the initial demonstration phase of the project. Perceived outcomes included indicators of progress that were shaped by diverse lived experiences, and envisioning the drone’s future potential.

#### Process: Maintaining the Vision During the Initial Demonstration Phase of a Feasibility Project

Participants described dissonance between what the drone was able to provide, what the community needed most, and what the future could hold. This dissonance was felt by both SC members and the OT. One participant shared,

this is the fun stuff that some of us get to do on the side of our desk, right? But the meat and potatoes of it is really not something that’s serving our community needs now.

[Justine, SC]

Natalie (SC) shared that staff also found it challenging to see the bigger picture and shared that she

would have to constantly remind [them] that we would have no problem receiving [laboratory] specimens from [nearby communities], they’re ten minutes away, but it’s the bigger picture, [the drone] wasn’t just intended to just do this route.

[Natalie, SC]

Some participants recognized this dissonance yet still saw value in the drone project. Even though it was possible to visit the pharmacy, putting the medication on the drone and following the established workflow was fundamental for project partners to get from where they were to where they wanted to be. One participant shared that,

rather than just go into the pharmacist, put it on the drone, take it off. Just to protect that process, which I thought was pretty good ingenuity, just to keep that idea of what we’re trying to do.

[Elise, SC]

#### Outcome: Perceptions of Progress That Are Shaped by Diverse Lived Experiences

Participants identified an array of indicators of the project’s perceived success. Many remarked on specific positive outcomes of the project, including forming relationships, making progress in the regulatory sphere, and other aspects of community and individual well-being, such as employment opportunities and completing medication deliveries. However, some struggled to see any immediate benefits.

The milestone of transporting real medications instead of surrogate packages was viewed by Thomas (OT) as a significant achievement. He shared,

I think the biggest milestone is that we actually did deliver a few medications in the drone. That was the concept, and we actually proved the concept, that it is something which is feasible*.*

[Thomas, OT]

Sydney (SC) also perceived the delivery of medical supplies as important and noted that it helped staff be more engaged and find purpose in their roles. She noted,

when we started to move the medical supplies on Tuesdays and Thursdays, it showed [local staff] there’s a real reason why we’re doing this, and that helped them get engaged more.

[Sydney, SC]

The diversity of lived experiences within the project contributed to varied perceptions of progress. Although some participants saw the delivery of real supplies as having a positive impact on themselves, staff, and community members, others did not. One participant shared,

I understand that it might be beneficial in areas where the roads are not accessible, but I feel like the amount of money that is spent on it, you would be better off driving in a vehicle.

[Rebecca, SC]

She later acknowledged,

what you’re doing right now, I feel like it is not really valuable to anybody, but I understand that you have to start at that smaller scale.

[Rebecca, SC]

Participants may have had difficulty seeing the drone’s positive impact because its service route was between 2 communities connected by road. However, other participants saw it differently: even though the route was short, the drone provided access to those who could not drive. Spencer (SC) shared, “we have clients that have no vehicles that rely on that drone.”

An important consideration also emerged when the drone delivery services that community members had begun to depend on stopped with the end of Phase I. Spencer (SC) offered insights on how the end of Phase I impacted community well-being. He shared that delivery of medications by the drone

really helped for people who don’t have vehicles within our communities, and some of the Elders, it’s delivered right to their door. I wish it would have continued because now I know those clients are going to be expecting the drone to drop it off for them.

[Spencer, SC]

Spencer (SC) also shared a frustration that highlighted discordance between the constraints of project funding models, project implementation, and First Nations cultural values. Spencer (SC) shared, “When you bring stuff to our communities you’re supposed to leave it, and you’re not supposed to take it back.” This frustration is embedded in a long history of government and academic research projects that are constrained to short-term funding timelines and lack continuity and sustainability. These types of projects can cause harm to communities and negatively impact relationships between First Nations and health system partners.

#### Outcome: Envisioning the Future Potential of Drones

Participants discussed partners’ improved understanding of the drone’s future potential as an important positive outcome of the project. For example, Elise (SC) said: “the big thing I heard is the possibilities of the drone project.” These ranged from further applications in health and wellness including the transport of traditional medicine, blood samples, or “even organs someday.” Other ideas included expanding the project to other First Nations communities and diversifying the drone’s capacity to assist in climate-change-related disaster relief or economic development opportunities.

One participant noted,

the situation especially on the Western side of Canada, regarding disaster recovery because of global warming, it’s a pretty significant problem. I think having contingency plans to use some of the drone solutions that we have for disaster support – it’s going to be needed for the foreseeable future.

[Christopher, SC]

Participants indicated that the drone’s utility could extend beyond medication delivery to facilitate the Stellat’en First Nation in pursuing their distinct priorities (health care, economic development, environmental monitoring, etc). Indeed, participants felt that “idea[s] for the drone [are] endless” and envisioned diverse future possibilities for the project. Although partners expressed innovative ideas for the future, Justine (SC) acknowledged distinctions between Nations and their priorities when considering how this project might be expanded to other First Nations in the future, and shared, “I wouldn’t say any one of our communities would look exactly as the other.”

## Discussion

### Principal Findings

Participants generally had positive experiences of being part of the DTI and highlighted the social aspects of this unique health innovation project. While technological challenges and benefits of drone use in health care settings have been widely discussed in the literature [19-21], we contribute an analysis of the social aspects and implications of integrating drone technology in rural and First Nation communities.

### Relationship-Building and Community Engagement Are the Foundation

Across all themes, deliberate efforts to cultivate relationships and community engagement were highlighted. Underpinning these efforts were trust and respect, factors which have been found to support efficient development and implementation of virtual health innovations in rural, remote, and Indigenous communities [22]. However, trusting relationships with communities were not built from scratch; rather, they had been cultivated over several decades between communities and Dr John Pawlovich, practicing rural family physician and project cosponsor. A unique characteristic of this project was preestablished, longitudinal relationships which enabled it to be implemented within a short timeframe and during a global pandemic.

Trusting relationships are critical in health projects with Indigenous peoples given the historical and contemporary injustices rooted in colonialism and white supremacy [23-25]. Centering Indigenous values within the project according to relational science models can support sustainable and equitable partnerships with Indigenous communities [26]. Youth engagement amplified support for the project, aligning with findings previously shown in community-led projects [27]. However, the role of youth extends beyond generating excitement; youth are the future of these communities. Exposure to technological innovations via the DTI embeds the project within communities longitudinally and inspires participation and leadership in the project by the next generation, or interest in science, technology, engineering, math, and medicine.

More broadly, public acceptance of drones is important as their use varies from espionage and military to parcel delivery [28]. The public may not typically associate drones with health care, but it has been shown that having knowledge about drones increases their acceptability [12,28]. As described in the literature, building trusting relationships and community engagement activities take time [29,30]. However, with a project manager, time and resources could be dedicated to facilitating continuous opportunities for engagement of the SC, OT, and communities. Moreover, the project manager could “meet people where they’re at*”* and engage them according to a flexible approach that respected individual and community capacity and ensured protocols for engaging with the Stellat’en First Nation were followed.

### Achieving Mutual Benefits and Imagining the Future

Participants benefited in various ways from involvement in the DTI at the individual (eg, enhancing medical delivery), communal (eg, strengthening relationships), and organizational (eg, leadership) levels. These findings align with depictions of previous health research partnership projects with Indigenous communities [31], describing benefits on different levels. Achieving mutual benefits relates to the concept of reciprocity: recognized as part of co-created projects in community-based health services, and an important cultural value in an Indigenous context [32].

Participants valued helping their communities, developing personal capacities, and having the freedom to be innovative. These are also reported as key elements of co-creation [32]. While environments supporting opportunities for creativity, growth, and mutual benefits encourage effective and engaged partnerships, procedures, policies, and organizational culture can impede engagement [33]. This was felt by some participants who did not feel supported by their organizations to build capacity within the DTI project.

Project champions reminded people of the DTI’s long-term vision and helped overcome short-term barriers such as perceiving a lack of immediate benefits of the project. This aligns with aspects of transformational leadership theory and the LEADS (Lead self, Engage others, Achieve results, Develop coalitions, and Systems transformation) framework, both Western perspectives that include inspiring, motivating, and empowering teams [25,34]. The LEADS framework is often used in health system contexts to describe strategic approaches to leadership and has been mapped onto Indigenous understandings of leadership [25]. This congruency reflects the true collaboration required within a novel, co-created innovation project.

Drone technology may also address uncertainties faced by rural and remote communities, which include the changing nature of the resource economy and the growing impact of climate change [35]. A drone may offer communities a tool to shape their futures and contribute to their economic well-being, including job creation and opportunities in areas beyond health care such as environmental monitoring, surveying, and natural resource exploration. Moreover, for Indigenous communities, where health is understood holistically, a drone may contribute to community well-being, aligning with the First Nations Perspective on Health and Wellness. In this model, health encompasses not only physical well-being, but also other factors including relationships and economic well-being, caring for the land and natural resources, as well as safety and emergency preparedness [36]. Participants shared how drones could be used according to communities’ distinct priorities now and in the future, and co-design of technology will enable the drone to be a fit-for-purpose tool suited to the unique realities of First Nations communities.

### Implications and Future Directions

Our findings highlight the importance of relationships within health care innovation projects with rural and First Nation communities that involve drones. Despite the tendency of rural and First Nation communities to be pushed to the edge of health care [22], this study similarly reframes and expands our notion of the edge. Instead of a place of exclusion, the edge in our context refers to the cutting edge—the forefront of innovation—where relational approaches and partnerships position communities to be active champions of projects that align with their development objectives. In a First Nations context, these notions contribute to broader goals of self-determination.

For the DTI Project Team, these learnings are essential to maintain a sustainable project that is accountable to the distinct priorities of the Stellat’en First Nation and informs approaches to forming partnerships with other rural and First Nation communities to expand the project in the future. The next phase of the DTI will involve implementing combined virtual pharmacy services and drone-based medication delivery in rural, remote, and Indigenous communities. The findings from this paper have directly informed our approach moving forward, reinforcing the importance of continuing in partnership using a relational, community-driven process. In collaboration with involved communities, we will co-create the service model, evaluate its impact, and document our findings in detail, from both operational and research perspectives.

The DTI Project Team and other project teams working in a health care innovation context will benefit not only from prioritizing relationship-building, but also from being able to understand and evaluate these partnerships. This may include establishing co-created definitions and indicators of success, partnership strength, effectiveness of leadership strategies, and trust-building that resonate with involved partners. These metrics will be crucial for project sustainability and scaling up the use of drones to other communities. The Partnership Pentagram Plus model previously used in health innovation projects could support future partnership and evaluation efforts for the DTI project, and other health care innovation projects with rural and Indigenous communities [10,37].

The implications of this work for academia more broadly are to exemplify disruption of conventional views of community-academia relationships as transactional and transient; instead supporting the notion that longitudinal partnerships that transcend the research life cycle [29] are not only beneficial, but essential for such work. The DTI’s focus on longitudinal relationships and mutual understanding is necessary for co-creating sustainable projects with Indigenous communities.

In a policy context, this work highlights the importance of relationship-building to support collaboration between industry partners, health care, and other sectors, communities, and First Nations rightsholders. However, bold, challenging, and innovative projects such as the DTI also depend on policymakers who can advocate for the value of co-creation, regardless of political leadership.

For health system leaders, this project illustrates how health technology can be integrated into the health care system in rural and Indigenous contexts. The insights described in this paper, together with an additional analysis of influencing factors of the DTI, will guide the development of an integrated drone network within the British Columbia health system (K Nouhi, unpublished data). This work can inform future efforts to expand drone technology to other Indigenous communities within and beyond the province of British Columbia.

### Limitations

Phase I was a 1-year proof-of-concept project that explored the feasibility of drone transport from the Village of Fraser Lake to the Stellat’en First Nation. When Phase I began, the drone had not yet been approved to transport medications (Transport of Dangerous Goods), and the Phase I evaluation primarily focused on perceptions and experiences of the Project Team to inform future phases. When approval was received to deliver medications, several community members received their medications by drone. As delivery of medication was not initially planned, we did not study the experiences of community members who used the drone’s services. Additionally, we did not explore the experiences of youth from communities. Future research is needed to understand how to optimally engage youth in health technology projects and how to evaluate its impact.

Interviews were conducted both individually and in groups, and the interview configuration was not explicitly considered in the analysis process (ie, all transcripts were analyzed in the same way). Group interviews may be influenced by what participants feel comfortable sharing, depending on their relationships with colleagues. Conversely, group settings can also deepen reflection and discussion, thereby enriching the findings.

Initial data analysis was conducted by researchers who were not involved in project planning, implementation, or data collection. This is generally disadvantageous for qualitative research; however, it afforded a “naïve approach” to the analysis that was not influenced by prior relationships or contextual understanding of the project. As AW and FH were not primed to interpret the transcripts in a particular way, the initial analysis enabled coauthors to contribute context and insight from their experiences within the project. Furthermore, the lead authors (AW, SL, and FH) do not have lived experience in a rural community. To mitigate this limitation, coauthors with various backgrounds were involved throughout the analysis and provided their perspectives on the results, and a first author positionality statement has been provided (Multimedia Appendix 4) to share how AW’s identity as a researcher and human being has shaped these findings.

Finally, the interview guide for this project was developed for a program evaluation, rather than a research study. For this reason, the questions were pragmatic and closed and were designed to capture pertinent information about the project for communities and funders. However, conversational-style interviews conducted by SL encouraged open and honest responses, in addition to the relationships that had been cultivated between SL and participants throughout the DTI project. This generated rich data suitable for this thematic analysis.

### Conclusions

There is promising potential for drones to address health inequities with the transport of medical supplies to rural and First Nations communities in British Columbia and beyond. These findings emphasize the importance of building trusting relationships between involved communities and partners, and the potential for drones to be used by communities for their distinct priorities within and beyond health care. This work highlights the relational foundation that is required for implementing and sustaining drone and other innovation projects in rural First Nations and non–First Nations communities. These outcomes will guide the co-creation of a health model of partnership evaluation specific to drone use in a Canadian context and highlight relational considerations in drone work.

## Supplementary material

10.2196/82720 Multimedia Appendix 1 Interview guide.

10.2196/82720 Multimedia Appendix 2 Survey development and data.

10.2196/82720 Multimedia Appendix 3 Project description.

10.2196/82720 Multimedia Appendix 4 Positionality statement.

10.2196/82720 Checklist 1 COREQ checklist.

## Abbreviations

COREQ: Consolidated Criteria for Reporting Qualitative Research
DTI: Drone Transport Initiative
LEADS: Lead self, Engage others, Achieve results, Develop coalitions, and Systems transformation
OT: Operational Team
SC: Steering Committee
TCPS 2: Tri-Council Policy Statement: Ethical Conduct for Research Involving Humans
UBC: University of British Columbia
