# Supplementary material for: Experiences and Perceptions Within a Co-Created Drone Transport Initiative With Rural First Nation and Non–First Nation Communities: Semistructured Interview Study
Source: J Particip Med. 2026 May 29;18:e82720. doi: 10.2196/82720 (PMC13220978; doi:10.2196/82720)
Supplement: Multimedia Appendix 1 [file jopm-v18-e82720-s001.docx]

Interview Guide

SL sent the following email to interviewees:

Thank you again for taking the time to provide your feedback. As discussed at the SC meetings, we hope to capture your feedback. This allows our project to ensure continuous improvement, as well as to capture lessons learned for the project reporting and future project planning.

The assessment is relatively informal and is meant to collect feedback on the project initiation, planning, implementation and reporting thus far:

- Any survey questions you would like to elaborate on in the interview?
- What went well for the project?
- What could we have done better?
- Do you think you were engaged to the best of your expertise throughout the project lifecycles? Was there anything UBC could do to support you or improve your participation experience?
- Do you feel supported by your organization at the senior and operational level to participate in the project? Are there suggestions on how we can engage your organization better?
- Anything you want to highlight in terms of engaging First Nations communities from your role in the project or experience in general?
- Are there other ways drone technology can improve rural and remote communities, including First Nations communities?
- (include any identified areas of focus by the interviewee)
- Anything else not covered in the interview that you would like us to include?

As part of our evaluation, we would like to record the Zoom meeting for transcription and systematic analysis with other participants. Any findings with your personal identifier (i.e. name or role) will be sent to you for review and approval before sharing. Please let me know if you have any questions or concerns when we connect.

I look forward to this meeting and thank you again for taking the time out of your busy schedules.
