# Supplementary material for: Experiences and Perceptions Within a Co-Created Drone Transport Initiative With Rural First Nation and Non–First Nation Communities: Semistructured Interview Study
Source: J Particip Med. 2026 May 29;18:e82720. doi: 10.2196/82720 (PMC13220978; doi:10.2196/82720)
Supplement: Multimedia Appendix 2 [file jopm-v18-e82720-s002.docx]

Survey Development and Data

The table below describes an overview of how representatives of three organizations (Rural Coordination Centre of BC, First Nation Health Authority and Stellat’en First Nation) were involved in the design of the project.

**Table S1.** Collaborator roles in project design.

| **Organization** | **Role** |
| --- | --- |
| **First Nations Health Authority** | - Reviewed methodology and committees to ensure they respected cultural safety and humility - Recommended to weave both Western and Indigenous ways of knowing into the evaluation |
| **Rural Coordination Centre of BC** | - Provided feedback on areas to emphasize - Recommended verbal consent rather than written consent |
| **Stellat’en First Nation** | - Confirmed survey tool, availability of technology, identified staff to support in-person survey collection, reviewed methodology and confirmed its feasibility with community - Recommended verbal consent rather than written consent - Requested interviews to be audio recorded |

**Table S2.** Project Strategic Partnership Assessment by Steering Committee.

| **Survey item** | **Responses (n = 9)** |
| --- | --- |
|  | mean ± SD [range] |
| **Project Participation Assessment** |  |
| 1. I am aware of the drone project's purpose and deliverables | 5.0 ± 0.0 [5-5] |
| 1. I believe this project has achieved its goals and purposes | 4.22 ± 0.67 [3-5] |
| 1. I think the drone project is considering my opinion and my organization's values while implementing the project | 4.44 ± 0.53 [4-5] |
| 1. I want to change the way we work together in this project | 3.22 ± 1.30 [1-5] |
| 1. I always know what is expected of me in this project | 4.11 ± 0.60 [3-5] |
| 1. I have access to sufficient information and support to complete my tasks in the project | 4.33 ± 0.50 [4-5] |
| 1. I believe drone technology can improve patients' access to care and advance health care service delivery | 4.78 ± 0.44 [4-5] |
| 1. Given the opportunity, I will participate in the next phase of the drone project again | 4.89 ± 0.33 [4-5] |
| **Principle 1 - Recognize and accept the need for partnership** |  |
| 1. There have been substantive achievements within the project partnership | 4.67 ± 0.50 [4-5] |
| 1. The factors associated with successful partnership are known and understood | 4.44 ± 0.53 [4-5] |
| 1. The barriers to a successful partnership are known and understood | 4.33 ± 0.71 [3-5] |
| 1. There is a clear understanding of when partners should work independently and collaboratively to achieve the project goals | 4.44 ± 0.73 [3-5] |
| **Principle 2 - Develop clarity and realism of purpose** |  |
| 1. The project partnership has a clear vision, shared values, and agreed deliverables | 4.89 ± 0.33 [4-5] |
| 1. I feel like my input and interests in my organization contribute to the joint aims and objectives of the project | 4.78 ± 0.44 [4-5] |
| 1. The joint aims and objectives of the drone project are realistic | 4.56 ± 0.53 [4-5] |
| 1. The reason why each partner is engaged in the partnership is understood and accepted | 4.67 ± 0.50 [4-5] |
| 1. We identified ‘small wins’ that leads to the long-term goal at the early stages of the partnership | 4.78 ± 0.44 [4-5] |
| **Principle 3 - Ensure commitment and ownership** |  |
| 1. There is a clear commitment to partnership from the most senior levels of my organization | 4.78 ± 0.44 [4-5] |
| 1. There is ownership in the required levels to contribute to the partnership within my organization | 4.78 ± 0.44 [4-5] |
| 1. I perceive mutual ownership of the project | 4.22 ± 1.01 [2-5] |
| **Principle 4 - Develop and maintain trust** |  |
| 1. The way the partnership is structured recognizes and values my organization's contribution | 4.22 ± 0.97 [2-5] |
| 1. Levels of trust within the partnership are high and can effectively address conflict, misunderstanding, or differences in opinion | 4.67 ± 0.71 [3-5] |
| 1. The partnership has succeeded in having the right people in the right place at the right time to advance the project | 4.56 ± 0.73 [3-5] |
| **Principle 5 - Create clear and robust partnership arrangement** |  |
| 1. Each partner's areas of responsibility are clear and understood | 4.67 ± 0.50 [4-5] |
| 1. There are clear lines of accountability for performance as a whole | 4.33 ± 1.00 [2-5] |
| 1. Operational partnership arrangements are simple, time-limited, and task-oriented | 4.67 ± 0.50 [4-5] |
| **Principle 6 - Monitor, measure, and learn** |  |
| 1. There are clear arrangements to ensure that monitoring and reviewing findings are, or will be, widely shared and disseminated amongst the partners | 4.56 ± 0.53 [4-5] |
| 1. Project updates and advancements are well communicated outside the partnership | 4.78 ± 0.44 [4-5] |
| 1. There are concise arrangements to ensure that partnership aims, objectives, and work arrangements are reconsidered and, where necessary, revised in light of feedback | 4.67 ± 0.50 [4-5] |

Note: All items were answered on a 5-point Likert scale in which 1 = strongly disagree and 5 = strongly agree.

**Table S3.** Project Assessment Operational by Operational Team.

| **Survey item** | **Responses (n = 9)** |
| --- | --- |
|  | mean ± SD [range] |
| **Project Planning Process** |  |
| 1. I am aware of the drone project's purpose and deliverables | 3.00 ± 1.80 [1-5] |
| 1. I believe this project has achieved its goals and purposes | 3.11 ± 1.36 [1-5] |
| 1. I believe the aims and objectives of the drone project are realistic | 3.11 ± 1.54 [1-5] |
| 1. I believe drone technology can improve patients' access to care and advance health care service delivery | 3.44 ± 1.88 [1-5] |
| **Project Goals and Outcomes** |  |
| 1. The factors that enable project success is understood and implemented | 3.22 ± 1.48 [1-5] |
| 1. The barriers of this project are understood and addressed | 3.11 ± 1.54 [1-5] |
| 1. Each partner's areas of responsibility are clear and understood | 3.44 ± 1.59 [1-5] |
| 1. My opinions and my organization's values are valued and implemented in the project | 3.33 ± 1.73 [1-5] |
| 1. I wish I was engaged earlier | 2.67 ± 1.41 [1-5] |
| **Organizational Commitment** |  |
| 1. I feel supported by senior leadership and my supervisor to participate in the project | 3.22 ± 1.56 [1-5] |
| 1. The right people in the right place at the right time from my organization were engaged to advance the project | 3.67 ± 1.66 [1-5] |
| **Participation** |  |
| 1. I feel like participating in this project was easy | 3.67 ± 1.12 [2-5] |
| 1. I understand when I can work independently to advance this project's goals | 3.56 ± 1.13 [1-5] |
| 1. I want to change the way we work together in this project | 2.78 ± 1.30 [1-5] |
| 1. Given the opportunity, I will participate in the next phase of the drone project again | 3.44 ± 1.51 [1-5] |
| **Information Sharing** |  |
| 1. I have access to sufficient information and support to provide input to the project | 3.11 ± 1.62 [1-5] |
| 1. I have access to sufficient training to complete my tasks as part of this project | 3.89 ± 1.69 [1-5] |
| 1. I feel like the right level of project updates and information are communicated to me | 3.38* ± 1.85 [1-5] |

Note: *n=8; All items were answered on a 5-point Likert scale in which 1 = strongly disagree and 5 = strongly agree.
