# Supplementary material for: Experiences and Perceptions Within a Co-Created Drone Transport Initiative With Rural First Nation and Non–First Nation Communities: Semistructured Interview Study
Source: J Particip Med. 2026 May 29;18:e82720. doi: 10.2196/82720 (PMC13220978; doi:10.2196/82720)
Supplement: Multimedia Appendix 3 [file jopm-v18-e82720-s003.docx]

Project Description

Project Initiation

In the spring and summer of 2020, partners were engaged, and a project concept was developed. An application was drafted for the TD Ready Challenge, an initiative that awards funding to innovative projects in Canada and the United States. The application was submitted in August 2020, and funding was received in December 2020.

Project Planning

In January 2021, partners and community were engaged. In May 2021, the Senior Project Manager was hired. In July 2021, a contract was signed with Drone Delivery Canada (DDC), a drone technology provider, for the lease of a drone for a one-year period. In July and August 2021, site reconnaissance was done, land was secured for use with the Stellat’en First Nation and the Village of Fraser Lake, and drone site infrastructure was established.

Project Implementation (Phase I)

Between September and December 2021, staff were hired and onboarded. A Student Logo and Naming Competition was held in September 2021. The project was implemented in October 2021 with a Phase I Opening Ceremony that was held in community. The drone’s flight path was short distance of 5 km (8-minute flight), and the drone could accommodate a payload of 10 lbs. The drone flew 14-16 times daily (M-F, 8:30am – 4:30 pm) at 200 ft above ground. Initially, the drone was only approved to transport mock medical supplies. However, in January 2022, the project received Transportation of Dangerous Goods approval from Transport Canada, which includes the transport of medications. In March 2022, the drone began transporting medication to members of the Stellat’en First Nation who chose to take part in the project.

Temperature protocol development and testing occurred between January and June 2022, and in May 2022, the project was approved by Transport Canada for Beyond Visual Line of Sight drone operations. Between July and September 2022, the project received Diagnostic Accreditation Program tabletop accreditation. Phase I concluded in October 2022 with a Closing Ceremony.

Project Roles and Organizational Structure

A full-time Senior Project Manager was hired to oversee the work. This was different from work that is typically done by universities, which may be overseen by a graduate student and/or postdoctoral researcher. The Senior Project Manager had project management expertise pertaining to partnership projects in her former roles at First Nations Health Authority and with Vancouver Coastal Health. The Senior Project Manager position was full-time on this project, which helped to build relationships and support the flexible engagement approach that was necessary for this project. The DTI Project Team was made up of the SC and the OT. The SC included individuals who occupied leadership positions in their respective organizations (Stellat’en First Nation, Carrier Sekani Family Services, Stellat’en First Nation Health Centre, Village of Fraser Lake, Northern Health, Fraser Lake Community Health Centre, Fraser Lake Pharmacy, LifeLabs) and were authorized to delegate resources in support of the project. The OT included individuals who were involved in the day-to-day operations of the project; individuals who worked in the Stellat’en First Nation and the Village of Fraser Lake and Operating Control Centre staff from Drone Delivery Canada who are based out of their headquarters in Ontario.

Community Engagement Efforts

Communities were engaged throughout the project in multiple ways. The Village of Fraser Lake took part in two Community Engagement meetings, one Mayor and Council meeting, and the Stellat’en First Nation took part in four community meetings, and one Annual General Assembly. Both communities participated in the Phase I Opening and Closing Ceremonies. School District 91 was engaged in a Drone Naming and Logo Contest (winning name: “Sky Medic” by Jasper, age 11). Fifty students ranging from kindergarten to grade 3 were reached through school field trips and presentations, and 60 students in grades 4-12 were reached through presentations.

Phase I project collaborators included the Stellat’en First Nation, the Village of Fraser Lake, the Faculty of Medicine and Faculty of Pharmaceutical Sciences at the University of British Columbia, the First Nations Health Authority, Carrier Sekani Family Services, the Rural Coordination Centre of BC, LifeLabs, and BC School District 91. Donors of the project include TD Ready Challenge, LifeLabs, Canadian Healthy Communities, Canfor, Air Canada, and donations from UBC Giving Day.
