# Supplementary material for: Experiences and Perceptions Within a Co-Created Drone Transport Initiative With Rural First Nation and Non–First Nation Communities: Semistructured Interview Study
Source: J Particip Med. 2026 May 29;18:e82720. doi: 10.2196/82720 (PMC13220978; doi:10.2196/82720)
Supplement: Multimedia Appendix 4 [file jopm-v18-e82720-s004.docx]

Positionality Statement

Audrey Warner, MPH Student

I come into this work with profound humility as a third-generation white settler of European ancestry. Having been educated within academic institutions that have inherently racist and colonial structures, I acknowledge how my personal and academic upbringing affects my worldview and how I do this work. As a current Master of Public Health student, my motivation for joining this project was to contribute positively to efforts that support the wellbeing and self-determination of First Nations communities. While I seek to accurately reflect the perceptions and experiences of those who were involved in the project, I understand my positionality is a limitation to this work. To meaningfully synthesize the findings of this project, I have shared back what I understood from the data to ensure it reflects the experiences and perceptions of those who were involved. Academia is a powerful machine that can facilitate transformative change but can also do incredible harm. This is something I have been aware of throughout my involvement in this project and seek to recognize this duality and leverage my position to facilitate transformative change. Academic manuscripts are valuable forms of knowledge-sharing within specific circles; however, this knowledge tends to be kept within these boundaries and not shared beyond the academic audience. It is important to recognize the value of other forms of knowledge and knowledge-sharing beyond the academic manuscript. Thus, in addition to this article, this knowledge will be shared in ways that are meaningful to host communities.
